# Gollop-Wolfgang Complex

**DOI:** 10.21699/jns.v6i1.492

**Published:** 2017-01-01

**Authors:** Oumarou Habou, Ibrahim Amadou Magagi, Harissou Adamou

**Affiliations:** University of Zinder, Niger

**Figure:**
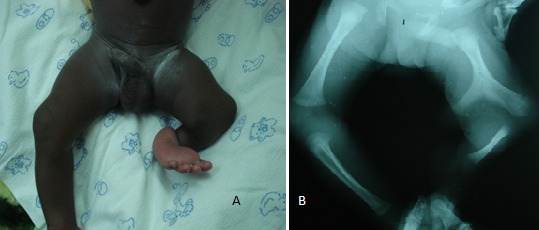
Figure 1: A) Left pelvic limb malformation. B) X-rays showing bifurcated fémur, tibia and patella agenesis

A full-term, 24-day-old male newborn was admitted with deformed left pelvic limb. He was born of a non-consanguineous couple without any medical history and no notion of congenital malformation in the family. The clinical examination revealed an anteromedial bony protrusion at the left knee, ipsilateral clubfoot and normal contralateral pelvic limb (Fig.1A). X-rays of the left pelvic limb showed a bifid femur with tibia agenesis and absence of the patella (Fig.1B). No further anomaly was found. Gollop-Wolfgang complex diagnosis was made.

Gollop-Wolfgang complex is a rare congenital orthopaedic malformation (1/106 live births) characterized by a distal femoral duplication and tibial agenesis associated or not with hand ectrodactyly [1,2]. The etiology of GWC is unknown and the surgical correction still difficult [1,3,4]. Excision of left sided bifurcated femur was proposed but the parents refused. Limb salvage treatment may be an alternative in this case, but would be impracticable in our context.

## Footnotes

**Source of Support:** Nil

**Conflict of Interest:** None
